# Chemotherapeutic properties and side-effects associated with the clinical practice of terpene alkaloids: paclitaxel, docetaxel, and cabazitaxel

**DOI:** 10.3389/fphar.2023.1157306

**Published:** 2023-05-09

**Authors:** Mário Sousa-Pimenta, Letícia M. Estevinho, Agnieszka Szopa, Mahnoor Basit, Khushbukhat Khan, Muhammad Armaghan, Manshuk Ibrayeva, Eda Sönmez Gürer, Daniela Calina, Christophe Hano, Javad Sharifi-Rad

**Affiliations:** ^1^ Department of Onco‐Hematology, Portuguese Institute of Oncology of Porto (IPO-Porto), Porto, Portugal; ^2^ i3S—Instituto de Investigação e Inovação em Saúde da Universidade do Porto, Porto, Portugal; ^3^ Mountain Research Center (CIMO), Polytechnic Institute of Bragança, Campus Santa Apolónia, Bragança, Portugal; ^4^ Department of Biology and Biotechnology, Agricultural College of Bragança, Polytechnic Institute of Bragança, Campus Santa Apolónia, Bragança, Portugal; ^5^ Chair and Department of Pharmaceutical Botany, Medical College, Jagiellonian University, Kraków, Poland; ^6^ Department of Healthcare Biotechnology, Atta-ur-Rahman School of Applied Biosciences (ASAB), National University of Sciences and Technology (NUST), Islamabad, Pakistan; ^7^ Department of Natural Sciences, Faculty of Science and Technology, Caspian University of Technology and Engineering named after Sh.Yessenov, Aktau, Kazakhstan; ^8^ Department of Pharmacognosy, Faculty of Pharmacy, Sivas Cumhuriyet University, Sivas, Türkiye; ^9^ Department of Clinical Pharmacy, University of Medicine and Pharmacy of Craiova, Craiova, Romania; ^10^ Department of Biological Chemistry, Université ď Orléans, Chartres, France; ^11^ Facultad de Medicina, Universidad del Azuay, Cuenca, Ecuador

**Keywords:** paclitaxel, docetaxel, cabazitaxel, toxoids, *Taxus brevifolia*, anticancer mechanisms

## Abstract

Over the years, many biological and synthetic agents have been explored and tested in attempts to halt the spread of cancer and/or cure it. Currently, several natural compounds have and are being considered in this regard. For example, paclitaxel is a potent anticancer drug that originates from the tree *Taxus brevifolia*. Paclitaxel has several derivatives, namely, docetaxel and cabazitaxel. These agents work by disrupting microtubule assembling dynamics and inducing cell cycle arrest at the G2/M phase of the cell cycle, ultimately triggering apoptosis. Such features have helped to establish paclitaxel as an authoritative therapeutic compound against neoplastic disorders. After the completion of compound (hemi) synthesis, this drug received approval for the treatment of solid tumors either alone or in combination with other agents. In this review, we explore the mechanisms of action of paclitaxel and its derivatives, the different formulations available, as well as the molecular pathways of cancer resistance, potential risks, and other therapeutic applications. In addition, the role of paclitaxel in hematological malignancies is explored, and potential limitations in the therapeutic use of paclitaxel at the clinical level are examined. Furthermore, paclitaxel is known to cause increased antigen presentation. The immunomodulatory potential of taxanes, alone or in combination with other pharmacologic agents, is explored. Despite terpene-alkaloids derivatives’ anti-mitotic potential, the impact of this class of drugs on other oncogenic pathways, such as epithelial-to-mesenchymal transition and the epigenetic modulation of the transcription profile of cancer cells, is also analyzed, shedding light on potential future chemotherapeutic approaches to cancer.

## 1 Introduction

The 20th century was a milestone in the history of human healthcare, marked by a continuous improvement in human longevity through reduced death rates from infectious diseases such as pneumonia, tuberculosis, and influenza. However, this progress came at the cost of an increased incidence of cancer (Jones et al., 2012). Paul Ehrlich coined the term chemotherapy and rendered pharmacognosy experimental. Posteriorly, the bombing of Bari harbor in World War II, which led to the spill of sulfur mustards and caused mucositis and granulocytopenia among survivors, allowed researchers to hypothesize the utility of mustard compounds in the treatment of lymphoproliferative malignancies and paved the way for the introduction of chemotherapy into clinical practice (DeVita and Chu, 2008). The progressive burden of neoplastic disorders and discovery of chemotherapeutic properties of natural, semi-synthetic, and repurposed chemical compounds ushered in an era of utilization of bioactive agents to fight lethal diseases. Paclitaxel (PTX) and its properties in neoplasms were first analyzed in the 1960s by the U.S. National Cancer Institute. This molecule was first obtained from the bark of *Taxus brevifolia* tree and yielded promising results. However, the technical, environmental, and economic challenges associated with the production and distribution of this chemotherapeutic agent prompted investigation into ways in which a more efficient production could be carried out through a well-established semi-synthetic process. Unfortunately, to date, combined efforts are still being carried out to develop a bioreactor (Gallego-Jara et al., 2020). After compound hemisynthesis was completed, the derivatives of the precursors obtained from *Taxus baccata* leaves were taken to clinical trials. This ultimately led to the approval of paclitaxel, and then some of its synthetic derivatives, either alone or in combination with other agents against solid malignancies. However, severe reactions from infusions were observed, resulting in the need to develop formulations with less severe side effects that do not require corticosteroids as a pre-medication (Desai et al., 2006a).

In this review, we explore the mechanism of action of PTX and its derivatives, the pharmacokinetics of the drug, its different formulations, cancer-related resistance mechanisms, and their indications and exposure hazards. Keeping the long-time side-effect of taxanes in mind, we analyzed the current evidence and proposed a pathophysiological perspective that may explain such an epidemiological correlation. Moreover, the effectiveness of taxanes as immunomodulatory agents has also been highlighted. Analogously, the extent to which taxanes and their derivatives can halt the growth of tumor cells is also reviewed. The role of taxanes has been established with the hallmarks of cancer like reversing metastasis, overcoming immunosuppression, inducing apoptosis, inhibiting angiogenesis, and restricting EMT.

## 2 Review methodology

A narrative review was performed. Electronic databases such as Medline/PubMed, ScienceDirect, Scopus, TRIP database, and Web of Science were verified to find both pre-clinical pharmacological/immunological studies and clinical trials regarding Taxol derivatives, using for searching the following MeSH terms: “Antineoplastic Agents/chemistry,” “Antineoplastic Agents/pharmacokinetics,” “Antineoplastic Agents/pharmacology,” “Apoptosis/drug effects,” “Clinical Trials as Topic,” “Drug Resistance,” “Neoplasm/physiology,” “Humans,” “Microtubules/drug effects,” “Mitosis/drug effects,” “Neoplasms/drug therapy,” “Taxoids/chemistry,” “Taxoids/pharmacokinetics,” “Taxoids/pharmacology,” “Paclitaxel,” “Paclitaxel/administration and dosage,” “Prostatic Neoplasms,” “Castration-Resistant/drug therapy,” “Docetaxel,” and “Taxoids/therapeutic use.” This study analyzed articles that explored the pharmacological, immunological reprogramming, and subcellular analysis of drugs in pre-clinical models using cell lines or animal models. It also assessed clinical trials that addressed the efficacy of these drugs in treating malignancies. Only studies published in English were included, and the most significant findings were summarized in tables and figures. The chemical formulas were validated using PubChem, while the taxonomy of plant species was confirmed using the World Flora Online.

## 3 Taxol derivatives: at a glance

**FIGURE 1:**
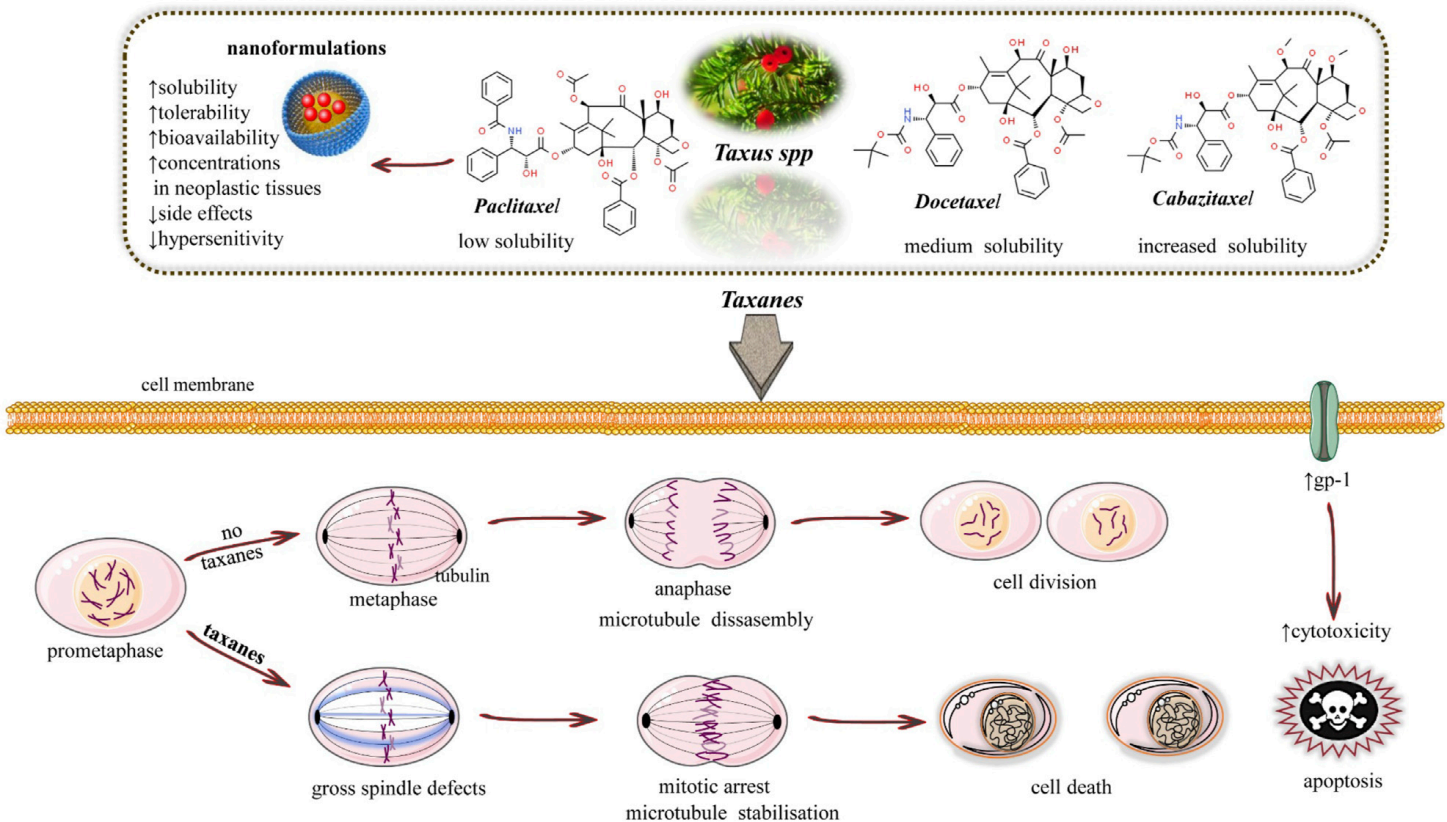
Illustrative scheme of the anticancer mechanisms of taxanes. They act as an antimitotic agent that promotes the assembly of tubulin dimers but inhibits the subsequent depolymerization process, herein stabilizing the microtubules (acting differently from Vinca alkaloids).

Taxol derivatives promote the assembly of microtubules and inhibit subsequent depolymerization, impairing the tubulin dynamics that foster the mitotic spindle assembly during interphase in mitosis (Figure 1).

### 3.1 Paclitaxel (PTX)

**FIGURE 2:**
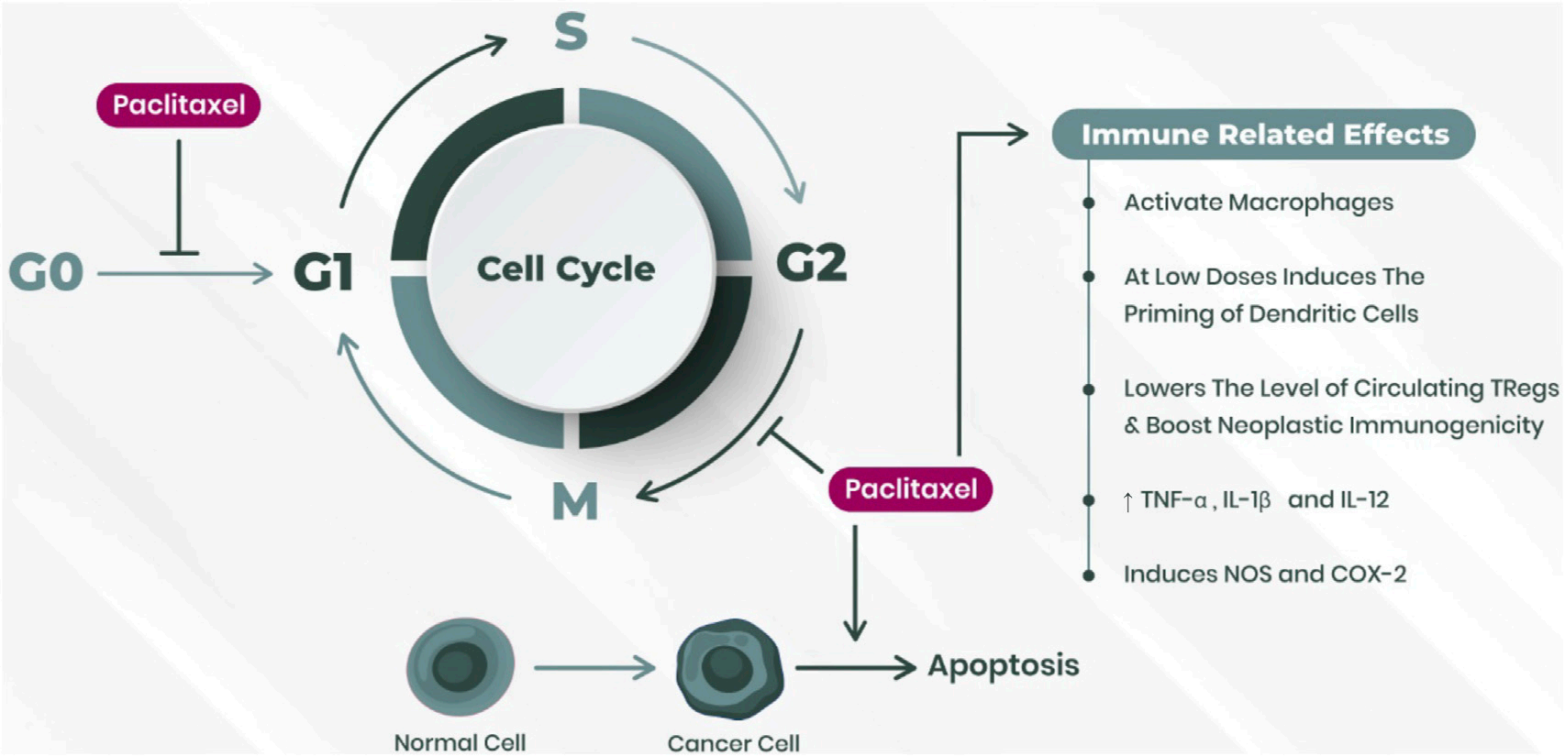
PTX influence on cell cycle regulation and immune responses. PTX hinders cell cycle progression at the G2 to M phase and from the G0 to G1 phase. It also initiates an immunogenic response toward tumor cells. Abbreviations: TNF, tumor necrosis factor; IL, interleukin; NOS, nitric oxide synthase; COX2, cyclooxygenase-2.

PTX was the first diterpene alkaloid isolated from the tree *Taxus brevifolia* in the 1960s. Dr. Jonathan Hartwell carried out this work at the scope of a National Cancer Institute U.S.A. (United States of America) screening program of antitumor properties of products derived from the plant kingdom. Although clinical trials with the diterpene alkaloid started in 1984, the high costs of extraction and purification associated with the depletion of *T. brevifolia* trees promoted a race toward developing a chemical semi-synthetic process. Given its complexity, a private company took over to attain completion (Gallego-Jara et al., 2020). It was not till the 1990s that the FDA (Food and Drug Administration) formally approved the drug for treatment of ovarian and metastatic breast cancer. This was followed by other neoplastic entities that benefitted from treatment with the agent (Kampan et al., 2015). The search for environmentally acceptable and economically profitable bioreactors to move away from the semi-synthetic route began after the fungus Taxomyces andreanae was identified as capable of producing PTX. After the first discovery, other fungal agents, mostly belonging to Ascomycetes, along with bacterial strains like *Bacillus cereus, Bacillus megaterium, Curtobacterium* sp*.*, and *Sphingomonas* sp*.* were reported as PTX producers. Although the assembly of the biosynthetic pathway in microbial heterologous hosts seems a promising strategy to lower production costs and increase the delivery of the chemotherapeutic agent, to date, no established bioreactor is in a working condition, and conventional production methods prevail (Flores-Bustamante et al., 2010). Overall, paclitaxel acts as an antimitotic agent that disrupts microtubule dynamics differently from vinca alkaloids. It promotes the assembly of tubulin dimers, leading to microtubule formation and stabilization. However, it subsequently inhibits their depolymerization, disrupting the dynamics of mitotic spindle formation and, ultimately, the cell cycle interphase. Exposed cells are blocked in the G2/M phase of the cell cycle, eventually undergoing apoptosis (Bates and Eastman, 2015) (Figure 2).

At low concentrations, PTX was shown to stimulate the priming of dendritic cells through TLR (toll-like-receptors) binding, ultimately fostering their maturation and function both in *in vitro* and *in vivo* studies. This eventually led to increased antigen presentation (Javeed et al., 2009). On the other hand, regulatory cells are responsible for maintaining immunological self-tolerance and are tendentiously increased in cancer patients, leading to a defective anti-tumor immunological response. PTX, inducing the expression of CD95 (cell-death receptor Fas), was shown to decrease the number of circulating regulatory T-cells, Tregs, at proportionally higher levels than in other lymphocyte populations (Javeed et al., 2009). Given the not stochastic and context-dependent role of PTX in immune cell-effector populations, drug repurposing in cancer as an immunotherapeutic adjuvant may be hypothesized. To this date, only some PTX chemo-resistance mechanisms have been brought to light. Upregulation of cytochrome P450 enzymes, namely, CYPC8 and CYP3A4 (common in neoplastic tissues), is an established resistance mechanism to drug action. Overexpression of P-glycoprotein is the major cause of diminished intracellular concentration of PTX, together with increased expression of the ABC (ATP-binding cassette) drug efflux pump, which also leads to increased extrusion of drug from neoplastic cells. ß-tubulin isoform preponderance may also render resistance to taxane therapy. For instance, ßIII-tubulin dimers exhibit a relatively low affinity to PTX and express elevated microtubular dynamics in the extreme ends of the mitotic fuse. They demand a higher ratio of tubulin to PTX-bound so that the cell cycle can be disrupted. In addition to such classical routes to cytotoxicity evasion, several cell-signaling disrupting non-coding RNAs and the overexpressed HIF-1α (hypoxia-inducible factor 1α) in neoplastic tissues have been associated with chemo-resistance. In the last case, HIF-1α signaling leads to a decreased pro-apoptotic mechanism and, in some malignancies, to phenotypic trans-differentiation. This is carried out through epithelial-to-mesenchymal transition (EMT) which is partially dependent on the expression of tumor growth factor β (TGF-β) and other cytokines (Maloney et al., 2020).

### 3.2 Docetaxel (DTX)

DTX was discovered by the French scientist Pierre Potier, who coined the semi-synthetic pathway of PTX synthesis using 10-DAB (10-deacetylbaccatin). In the abovementioned process, an intermediate, DTX, was found. Docetaxel binds ß-tubulin dimers in a 1:1 stoichiometric ratio, exhibiting a stronger dynamic instability using its inhibitory effect in tubulin depolymerization. Moreover, the drug exhibits better solubility and bioavailability (Xue et al., 2020). Docetaxel shows significant activity against prostate tumors. It has anti-neoplastic activity as it inhibits microtubular depolymerization and attenuates the expression of BCL-2 (B cell lymphoma 2 protein), and BCL-x genes. As a result, the cell cycle is arrested at G2/M phase, triggering a cascade of reactions that induce apoptotic cell death (Pienta, 2001). Beyond interfering with microtubular dynamics, docetaxel also induces cytoskeletal changes in malignant cells, overall contributing to a decreased proliferation, invasion and migration of cancer. Docetaxel-based chemotherapy is given to patients with gastric cancer. When combined with cisplatin, trials have reported a response rate of 55%, with a median overall survival of 9 months (Roth and Ajani, 2003). Docetaxel is usually given intravenously. The dosage and frequency are determined as per the type and size of the tumor. It can be administered alone or given in combination with other drugs, being of regard that docetaxel has increased side effects in patients whose liver function is abnormal (Fulton and Spencer, 1996).

### 3.3 Cabazitaxel (CTX)

CTX displays a poor affinity for ATP-dependent drug efflux pump P-glycoprotein (multidrug-resistant protein), herein displaying an advantage toward other taxanes. The drug displays bioactivity both in docetaxel-sensitive and -resistant cancers (preclinical studies and clinical trials), being approved by the FDA in 2010 in combination with prednisone for treatment of patients with hormone-refractory metastatic prostate cancer who have previously undergone treatment with docetaxel-containing regimens (Galsky et al., 2010). Just like other taxanes, cabazitaxel also works by binding to the microtubules. This prevents cellular mitosis and stabilizes the tumor cells. As a result, the cells do not divide. Cabazitaxel works by inhibiting androgen receptors by binding to the microtubules and microtubule-associated motor protein dynein. As a consequence, androgen receptor nuclear translocation is prevented. The formula for cabazitaxel is C_45_H_57_NO_14_. It is the second-generation derivative of taxanes following docetaxel. It is synthesized from 10-deacetyl baccatin III into a single diastereomer which is found in many Taxus species. Cabazitaxel depicts considerable anti-tumor activity in castration-resistant prostate cancer cell lines along with mouse models. Due to the over-expression of gp-1, cabazitaxel retains cytotoxicity in docetaxel-resistant cell lines. Compared to the other taxanes, cabazitaxel is soluble in water and able to penetrate through the blood–brain barrier, herein achieving the central nervous system (Galsky et al., 2010). In the clinical setting, docetaxel and prednisone are approved for the treatment of metastatic castration-resistant prostate cancer (mCRPC) (Tsao et al., 2014a).

## 4 Medical properties of taxanes

Taxol has properties that inhibit or halt tumor growth, including both solid and haematologic malignancies. PTX injections are administered to treat advanced stages of breast, ovary, non-small cell lung cancer, and Kaposi’s sarcoma. High doses of PTX can cause mitotic arrest at the G2/M phase, while low dosage induces apoptosis at the resting stage G0. Programmed cell death can also occur at the G1/S phase through the activation of Raf-1 kinase or p21/p53. The pathway is determined through the concentration of the dose and the neoplastic tissue intrinsic phenotype. On the other hand, PTX is also a potent drug when it comes to treating other diseases than cancer as well, accordingly to preclinical models. A low dose of PTX can treat non-cancer diseases like renal and hepatic fibrosis, skin disorders, inflammatory diseases, and coronary artery restenosis, mainly through modulation of TGF-β signaling pathway (Derynck and Zhang, 2003). A low dose of PTX (0.3 mg/kg) twice weekly is effective in reducing tubulointerstitial fibrosis in mouse models. In unilateral obstruction models, PTX proved to have a reno-protective role. Symptoms of pulmonary fibrosis were also reduced in rats administered with PTX (0.6 mg/kg/d) (Tsukada et al., 2013). Traumatic impacts can induce axonal damage in the central nervous system and lead to disabilities. In a study conducted by Sengottuvel and Fischer (2011), it was demonstrated that a low dosage of PTX can cause regeneration of neurons as it causes the stabilization of microtubules in the abovementioned cells. It was also observed that the rate of infiltrating macrophages was reduced, and glial scar formation was delayed. Overall, a dose of 256 ng/day promoted axons re-growth in rodents suffering from spinal injury (Hellal et al., 2011). Recent studies have also shown that the use of PTX is able to reduce vascular restenosis at a dose of 175 mg/mm^2^ (Tepe et al., 2007).

### 4.1 Taxanes reversing metastasis

PTX was first tested for its antitumor efficacy in lung cancer. A phase II trial was designed for non-small-cell lung cancer and was promoted to compare the efficacy of Taxol, merbarone, and piroxantrone in patients with stage IV metastasis. A total of 25 patients were enrolled in the Taxol group and 47 enrolled in the groups allocated to different drugs. In the Taxol group, patients underwent PTX infusion of 250 mg/m^2^ every 3 weeks. After 1 year, the survival rate was 41.7%, 21.6%, and 22.6% in patients treated with Taxol, merbarone, or piroxantrone, respectively, while the median survival times were 24.1, 19.9, and 29.3 weeks, respectively (Chang et al., 1993). Currently, PTX remains a prominent agent targeting metastasized squamous non-small-cell lung cancer, with the combination of nanoparticle albumin-bound [nab] PTX, a platinum agent, and the PD-1 inhibitor pembrolizumab regimen supplanting the previous frontline monotherapeutic approach consisting of either platinum-based chemotherapy or pembrolizumab. This lead to an increased response rate, free survival, and survival rate (Paz-Ares et al., 2018). In patients without metastases but locally advanced non-small-cell lung cancer (T2bN0, with a tumoral diameter higher than 4 cm), adjuvant chemotherapy with carboplatin and PTX may also be considered (Remon et al., 2021).

DTX, together with trastuzumab, was tested in a study by Baselga et al. (2012) which included 808 patients with HER2-positive metastatic breast cancer. Some patients also received treatment with docetaxel, trastuzumab, and pertuzumab. The results revealed a progression-free survival to be 12.4 months in the control group (docetaxel and transtuzumab with placebo) per comparison to 18.5 months in the triple therapeutic aproach group. Interim analysis revealed the overall survival trend to be favorable for pertuzumab plus trastuzumab plus docetaxel. This combination, as compared to the placebo, prolonged the progression-free survival significantly (Baselga et al., 2012). For inhibiting breast cancer metastasis, a docetaxel-loaded shrapnel nanodelivery system coupled with a matrix metalloproteinases-sensitive copolymer was properly developed. The drug was loaded on the liposomes and a structure of 113.3 ± 2.7 nm was created. It was observed that the drug was released simultaneously in the tumor micro-environment along with MMPs and reductive glutathione. This formulation significantly increased drug distribution in mice, with a higher effectivity and apoptosis induction in those ones having pulmonary metastasis (Xu et al., 2015).

CTX, as an anticancer drug, was loaded to form polymeric micelles, and their anti-metastatic potential was assessed both *in vivo* and *in vitro*. The average diameter of the micelle was 50.13 ± 11.96 nm, and the introduction of this drug had little effect on the viability of 4T1 cells, but it did have a strong effect on cell migration. The drug was posteriorly injected intravenously, and it resulted in 71.6% of tumor inhibition as well as 93.5% reduction in the lung metastasis of breast cancer in orthotopic rodent models (Zhong et al., 2017).

### 4.2 Taxanes overcoming immunosuppression

The first trial addressing PTX monotherapy in Kaposi´s sarcoma patients with HIV infection consisted of the infusion of 135 mg/m^2^ of PTX over 3 h every 3 weeks, which increased at each infusion of around 20 mg/m^2^ upon tolerability (granulocyte counts must remain above 1,000 leucocytes/µL). In that trial, patients with severe immunosuppression were included (mean CD4^+^ count at diagnosis of 16/µL), both with and without antiretroviral therapy. Around 65% of the patients achieved partial response (13 out of 20 enrolled) after an average of six cycles. Among responders, the median Kaplan–Meier progression-free survival rounded 34 weeks, and the median time to progression after discontinuation of chemotherapy was 10 weeks (SavilleLietzau et al., 1995). In disseminated or locally aggressive Kaposi’s sarcoma, PTX is still used in combination with a liposomal formulation of anthracycline as a chemotherapeutic regimen (Lebbe et al., 2019).

Adoptive cell therapy (ACT) is a promising strategy for treating cancer. However, this immunotherapeutic approach often leads to the accumulation of myeloid-derived suppressor cells (MDSCs) in the tumor microenvironment, which can counteract the desired immunogenic response. To overcome the immunosuppressive effect of MDSCs in ACT, docetaxel was used in a murine colon and breast cancer model to downregulate the recruitment and overall increase of MDSCs in the neoplastic microenvironment, thus boosting the effects of ACT mediated by T cells (Hu et al., 2020).

CTX is a second-generation semisynthetic taxane that is used to bypass the resistance induced by docetaxel and paclitaxel. The drug is administered by solubilizing it in polysorbate and ethanol. Cabazitaxel has also been assembled in nanoparticles and delivered in the form of a chemo-immunotherapeutic delivery system. The nanosystem works best in suppression of the growth of tumors and by remodeling the sensitivity of cancer cells toward chemotherapeutic drugs. All in all, the use of cabazitaxel is a good strategy for chemo-immunotherapy against solid prostate cancer tumors (Shi X. et al., 2022). Moreover, cabazitaxel is also known to promotes an inflammatory tumor microenvironment by inducing the activation of TLR3 in murine models (Deveci Ozkan et al., 2022).

### 4.3 Taxanes and induction of apoptosis

PTX stimulates the apoptotic modulating genes to promote programmed cell death of tumor cells. This is also interlinked with the transcription of genes that cause inflammation, DNA-damage response proteins, and cytokines that play a role in cellular proliferation. The rate of apoptosis of tumor cells is dependent on the time of exposure as well as the concentration of the drug. For instance, a concentration of 10 nM directly induces cell death through S phase induction without going through arresting at the mitotic stage, accordingly to *in vitro* assays (Chang et al., 1993).

The ability of DTX and vinorelbine to induce apoptosis of human prostate cancer cells was examined initially in two different LNCaP cell lines, C-33 and C-81. Vinorelbine and docetaxel have different binding sites on microtubules, which then trigger activation of distinct proapoptotic pathways (Zelivianski et al., 2003). Marked cell apoptosis and G2/M phase arrest were observed after treatment with docetaxel. High-dose (0.1 μM) docetaxel- and paclitaxel-treated cells resulted in a G2/M arrest, followed by generation of polyploidy or apoptosis; however, low-dose (0.01 μM) treatment induced apoptosis without G2/M arrest. These results suggest that, following the activation of NF-κB by docetaxel, apoptosis is elicited through a mitochondria-dependent pathway (Geng et al., 2003).

CTX is a taxane drug that can induce apoptosis or autophagy by inhibiting the phosphorylation of PI3K/Akt/mTOR and is effective in some drug-resistant tumors. It was demonstrated that cabazitaxel is highly toxic to hepatocellular carcinoma cell lines in a time- and dose-dependent manner by inducing G2/M phase arrest and apoptosis *in vitro*. Mechanistically, cabazitaxel induced hepatocellular carcinoma cells G2/M phase arrest via the Cdc25C/Cdc2/cyclin B1 pathway and apoptosis through the Bcl2/PARP pathway, consistent with its effect on other cancer cells (Chen et al., 2018).

### 4.4 Taxanes and inhibition of angiogenesis

PTX is known for exhibiting anti-angiogenic effects. At a low concentration of about 6 mg/Kg (intra-perithoneal), a considerable decrease in the vascular endothelial growth factor (VEGF) was observed in mouse models (Klauber et al., 1997).

Gastric cancer cell lines (BGC-823) were exposed to metronomic concentrations of DTX for 144 h. The abovementioned drug caused a reduction in the secretion of VEGF by BGC-823, which means that it could restrict the angiogenic potential of cancer (Wu et al., 2011).

In cellular models of glioma, cabazitaxel disrupted filamentous actin cytoskeleton dynamics and abrogated tumor-induced angiogenesis. These findings raise awareness not only about the cytotoxic effects of the drug but also about its potential to contain the spreading of cancer by reducing invasion and migration (Ghoochani et al., 2016).

### 4.5 Taxanes, molecular cascades, and targeted signaling pathways

Overall, paclitaxel inhibits the activation of Akt (downstream effector of the PI3K pathway), thus reducing the phosphorylation of mTOR and decreasing cell proliferation at the expense of increased cell death. The drug also activates C-Jun N-terminal kinase (JNK), ultimately boosting cell death (Rakovitch et al., 1999).

DTX can affect many signaling pathways and molecular pathways in cancer, which are involved in survival and growth of cancer. The mechanism of action of docetaxel involves the disruption of microtubule dynamics and interfering with cell division. This leads to apoptosis and cell cycle arrest. Docetaxel mainly targets molecular pathways like Akt/mTOR and NF-kB (Chen et al., 2014).

CTX downstream signaling is associated with the induction of apoptosis, activation of cell cycle checkpoints, and inhibition of signaling pathways like AKt/mTOR. It is also able to reverse EMT in the treatment of prostate cancer (Huo et al., 2016).

### 4.6 Taxanes and epigenetic changes

The epigenetic regulation of the genome can modulate the transcriptional profile of cancer cells, which is highly dependent on DNA methylation, histone modifications, and non-coding RNA molecules. This phenomenon, by changing the expression of tumor suppressor or oncogenic genes and their promoters and/or enhancers, is involved in cancer initiation, progression, and even chemoresistance. In experimental models of breast cancer, exposure of MCF-7 cells to paclitaxel in a low-concentration gradient induction method (aimed at promoting chemoresistance) showed an overall increase in DNA methylation pattern (Shi et al., 2020). To overcome this chemoresistance, the co-exposure of breast cancer cell lines to taxanes and phenethyl isothiocyanate (an epigenetic agent) was able to restore chemosensitivity (Liu et al., 2013).

Similarly, pre-treatment of DU145 prostate cancer cell lines, which were resistant to docetaxel and cabazitaxel, with 5-azacytidine (a hypomethylating agent) induced chemosensitivity to cabazitaxel (Ramachandran et al., 2016). Overall, evidence suggests that taxanes may impact on epigenetic regulation in cancer cells. The combination of taxanes with epigenetic modifier agents may, soon, overcome chemoresistance; and clinical trials in this regard are needed.

### 4.7 Taxanes and their role in EMT

The epithelial-to-mesenchymal transition (EMT) is a biological process in which epithelial cells lose their polarity and adhesion properties, resulting in the downregulation of epithelial markers and the upregulation of mesenchymal ones. Although EMT is highly implicated in embryonic development, tissue differentiation, and wound healing, it also occurs in neoplastic tissues. EMT frequently depends on the TGF-β signaling pathway and contributes to the increased invasiveness and metastasis of cancer cells (Dongre and Weinberg, 2019).

Relapse is an important issue faced during the treatment of breast cancer patients. Blocking EMT has been reported to suppress PTX-induced cancer stem cell (CSC) properties in cancer cell lines. Indeed, blocking TGF-β signaling reduces PTX-induced EMT and CSC-like characteristics in experimental in vitro assays of breast cancer (Park et al., 2015). Overtime treatment of neoplastic cells with sublethal doses of taxanes, beyond resistance, may lead to EMT. The exposure of colorectal cancer cell lines to sublethal doses of paclitaxel fostered an epithelial-to-mesenchymal transition, which was antagonized by all-trans retinoic acid (ATRA) co-treatment. ATRA was able to reduce EMT by upregulating gap junctions in cancer cells, while simultaneously downregulating NF-κB (Shi et al., 2019).

Clinical trials are warranted to adjust the dosage of taxane to patients’ pharmacokinetic profiles. Exploring chemoresistance and metastization potential mitigation strategies with combinatorial approaches with other drugs (such as ATRA) may also improve outcomes in the clinical setting.

Table 1 summarizes the most representative data of taxane efficacy in cancer.

**TABLE 1:** Approaching the role of taxanes in cancer pathophysiology.

Type of taxane	Immunomodulation	Apoptosis induction	Effects on angiogenesis	Targeted molecular cascades	Epigenetic changes	Effects on EMT	Ref
PTX	Stimulates the priming of dendritic cells; Activates macrophages; Induced expression of CD95; Decreases T regulatory cells		↓VEGF	p38 mitogen-activated protein	↑Raf-1	↓TGF-β	J. Remon et al. (2021)
		↑DNA damage		JNK	↑p21		Xu et al. (2015)
		↑inflammatory cytokines		TLR-4	↑p53	↓ALK5	Zhong et al. (2017)
		↑cell cycle arrest					
DTX	Increases the number of tumor-infiltrating T cells in neoplastic tissues; Enhances interferon signaling	↑cell cycle arrest in G2/M	↓TSP-1/VEGF	Disruption of microtubular dynamics		↑epithelial markers	C. Lebbe et al (2019)
							Tepe et al. (2007)
		↓NF-κB		AKT/mTOR NF-kB	↑genes involved in apoptosis	↓mesenchymal markers	Chang et al. (1993)
							Ma Z. et al. (2022)
CTX	Promotes an inflammatory tumor microenvironment by inducing the activation of TLR3 in mCRPC	↑autophagy	↓PAI ↓Maspin ↓TSP1	AKt/mTOR Disruption of microtubule dynamics	*In vitro* assays with DU145 cell line: pre-exposure to 5-AZA was able to restore chemosensitivity	Reversal of cancer cell migration	J. Remon et al. (2021) Ozkan et al. (2022)
		↓PI3K/Akt/mTOR					
		Phosphorylation					
		↑G2/M phase arrest					
		↓Cdc25C/Cdc2/cyclin B1					

		↓ BCL-2/↑ c-PARP					

Abbreviations and symbols: ↑stimulation; ↓ inhibition; epithelial-to-mesenchymal transition (EMT); vascular endothelial growth (VEGF), thrombospondin-1/vascular endothelial growth factor; Jun N-terminal kinase (JNK); Raf-1, proto-oncogene, serine/threonine kinase (Raf-1); transforming growth factor-β (TGF-β); deoxyribonucleic Acid (DNA); activin receptor-like kinase 5 (ALK5); phosphatidylinositol-3-kinase (PI3K)/Akt and the mammalian target of rapamycin (mTOR); thrombospondin 1 (TSP1); toll-like receptor 4 (TLR4).

## 5 Treatment regimens of taxanes: Data from clinical trials

Given its incidence and social awareness, breast cancer stayed on the frontline of neoplasms in which taxanes’ bioproperties were more prematurely and intensively assessed. In an early trial, a population with equally distributed pre-menopausal and post-menopausal status only including pre-treatment cases (chemotherapy with or without radiotherapy and/or hormonotherapy) with metastatic disease (skin 36%, lymph nodes 34%, bone 42%, and visceral 97%) underwent PTX 135 mg/m^2^ versus 175 mg/m^2^ infusion over 3 h every 3 weeks. Histologically, 28% were negative for estrogen and progesterone receptors, while 38% were positive. The overall response rate was 27%, with 27 partial responses and three complete responses. Drug-induced peripheral neuropathy presented a dose-dependent onset, affecting 61% of patients in the higher-dose group and 34% of those in the lower-dose group (Spielmann, 1994). Considering the promising results of the drug, duplets of PTX with doxorubicin were started in an attempt to achieve better clinical outcomes. Between 1993 and 1995, on behalf of the Eastern Cooperative Oncology Group, patients with advanced breast adenocarcinoma (metastasized or locally advanced), who had undergone prior chemotherapeutic regimens (containing neither taxanes nor doxorubicin) in a time frame not inferior to at least 6 months, were enrolled in a trial comparing PTX (175 mg/m^2^/24h), doxorubicin (60 mg/m^2^), or a combined regimen (PTX 150 mg/m^2^/24h, doxorubicin 50 mg/m^2^, and prophylactic granulocyte-colony stimulating factor) with infusions every 3 weeks. Patients were randomized, and the arms were matched for race, estrogen receptor status, performance status, regimens, and timeframe of the appliance. Once progression occurred in single-agent groups, patients were crossed over. Overall, results showed that the combinatory regimen versus sequential single-agent treatment improved the overall response rate (47% versus 36% and 34% for patients in doxorubicin and PTX, respectively) and increased time to treatment failure (median time of 8 months versus 5.8 and 6 months for patients in doxorubicin and PTX, respectively), although not significantly affecting the overall survival and quality of life of patients (Sledge et al., 2003). To date, taxanes remain an efficient strategy in the chemotherapeutic approach to metastatic breast cancer (MBC). An early phase II trial in MBC patients who were positive for human epidermal growth factor 2 (HER-2) and naïve to chemotherapeutic approaches demonstrated the superiority of the combinatorial trastuzumab and docetaxel regimen. The study showed an increase in overall survival by more than 8 months (31.2 versus 22.7 months for the combinatorial regimen and docetaxel alone, respectively) and time to disease progression (11.7 versus 5.7 months for the combinatorial regimen and docetaxel alone, respectively) (Marty et al., 2005). In patients with HER-2 positive MBC, the first-line therapeutic approach typically involves a triplet combination of pertuzumab, docetaxel, and trastuzumab. In the phase III CLEOPATRA trial, this triplet combination increased median progression-free survival by more than 15 months and progression-free survival by more than 6 months compared to docetaxel and trastuzumab alone. Overall, the median OS reached 56.5 months in the triplet group compared to an average of 49.3 months (Swain et al., 2015). In metastasized triple-negative breast cancer (mTNBC), tumors lack estrogen receptors, progesterone receptors, and amplification of HER-2, and the addition of immune-checkpoint inhibitors (either alone or combined with taxanes) is recomendend in patients where more than 1% of neoplastic cells express PD-L1 (assessed by immunohistochemistry) or when there is no overexpression of PD-L1 and gBRCAm-wild-type (Gennari et al., 2021). Herein, a phase III trial included patients with irresectable mTNBC, who were randomized to nab-PTX (at 100 mg/m^2^ at days 1, 8, and 15 of each 28-day cycle) with or without atezolizumab (every 2 weeks) till progression or intolerance, and performed a hierarchical analysis in the intention to treat population and in the PD-L1 positive population. Globally, atezolizumab plus nab-PTX increased progression-free survival and survival rate in the ITT group (although, in the former case, it was not in a statistically significant pattern). In patients whose cells expressed PD-L1 at a higher rate than 1%, the combinatorial regimen increased OS significantly (25 versus 15.1 months).

Besides the paradox of the chemotherapeutic approach to advanced breast cancer, taxanes are also approved in the adjuvant approach to the early-stage disease. A clinical trial demonstrated that in the early stages right after surgery (up to T_2_-T_3_, N_0_-N_1_, and M_0_), addition of PTX to doxorubicin, followed by administration of cyclophosphamide, methotrexate, and fluorouracil (CMF), significantly improved the relapse-free survival compared with doxorubicin standalone followed by CMF. At 7 years of follow-up, the combination of PTX and anthracycline yielded a 76% vs*.* 69% of relapse-free survival rate and 85% vs*.* 82% of overall survival in the combinatorial versus anthracycline-based regimens (Gianni et al., 2009). Moreover, PTX is also approved as an adjuvant chemotherapeutic agent in early-stage HER2-positive breast cancers in combination with trastuzumab (Tolaney et al., 2015) or the adjuvant approach to non-invasive and resectable triple-negative breast cancer in combination with platinum agents (Yu et al., 2020).

Pivotal trials of PTX in ovarian cancer addressed its role as a single agent in heavily pre-treated patients with refractory disease when administered in a dose of 135 mg/m^2^ every 3 weeks upon intolerance or progression. Among 68 enrolled patients, 15% showed partial response (whose duration ranged from 8 to more than 23 months), and 40% of patients presented a stable disease (with a median duration of response of 6.4 months) (Uziely et al., 1994). The current European orientations still advocate that in patients with granulosa cell tumor (at least stage IC2), after debulking, adjuvant chemotherapy is recommended with bleomycin, etoposide, and cisplatin (BEP) or with carboplatin and PTX. In the same way, in Sertoli–Leydig cell tumors (at least stage IB or IA if the neoplasm is poorly differentiated), after surgery/debulking, adjuvant chemotherapy with BEP or carboplatin and PTX is also recommended (Ray-Coquard et al., 2018).

The search for the role of Taxol derivatives in hematological malignancies has also been initiated, mainly focusing on relapsed or refractory non-Hodgkin lymphoma. In early trials, as a single chemotherapeutic agent, PTX achieved modest overall response rates, that ranged from 15% to 23% (Casasnovas et al., 2000) (Kahl et al., 2005), herein being disregarded due to classical chemotherapeutic regimens that target these malignancies and display better response rates and outcomes. In addition to the initial results, an *in vitro* preclinical work was conducted using a cell line derived from a patient with diffuse large B cell lymphoma who was resistant to doxorubicin. Results showed promising activity of PTX and docetaxel in the setting of anthracycline resistance acquisition in this lymphoproliferative disorder (Wu et al., 2014) (Table 2).

**TABLE 2:** Role of taxanes in the therapeutics of different cancers.

Tested taxane	Type of cancer	Dose	Patients enrolled	Outcomes
PTX	Lung cancer	250 mg/m^2^ every 3 weeks	25 patients enrolled in the paclitaxel group and 47 in groups with different drugs (merbarone and piroxantrone)	After 1 year, the survival rate was 41.7%, 21.6%, and 22.6%, respectively
Chang et al. (1993)				
PTX Monotherapy	Kaposi´s sarcoma in patients with HIV	135 mg/m^2^ of PTX over 3 h every 3 weeks	13 out of 20 enrolled	65% of the patients achieved partial response after an average of six cycles
Saville et al. (1995)				
Docetaxel	Metastatic breast cancer	100 mg/m^2^ versus	51 patients enrolled	The response rate registered was of 40% for 75 mg/m^2^ versus 63% for the 100 mg/m^2^ dosing regimen.
ME Trudeau et al. (1996)		75 mg/m^2^ every 3 weeks		
Docetaxel	Advanced gastric carcinoma	100 mg/m^2^ of docetaxel once every 3 weeks	37 patients with a median age of 59 years	Eight of the 33 evaluable patients (24%) achieved a partial remission. Eleven patients achieved stable disease
A Sulkes et al. (1994)				
Cabazitaxel	mRCPC	25 mg/m^2^	378 enrolled in cabazitaxel group and 377 in the mitoxantrone group	For a median follow-up higher than 2 years, the odds ratio of survival for the cabazitaxel treated group was of 2.11 (CI 95% 1.33–3.33).
Bahl et al. (2013)				

The National Cancer Institute of Canada Trials Group (NCIC-CTG) carried out a study regarding the toxicity as well as the efficacy of the DTX drug with metastatic breast cancer (MBC). A total of 51 patients were enrolled in the study. The docetaxel was administered each 3 weeks at a dose of 100 mg/m^2^ or 75 mg/m^2^. Almost 60% of patients developed hypersensitivity reactions, with the administration of H1 and H2 blockers preventing them. The response rate observed at 75 mg/m2 was 40%. However, the response rate was 63% for patients receiving the dose of 100 mg/m2 (Trudeau et al., 1996). Analogously, thirty seven patients with the diagnosis of gastric carcinoma and a median age of 59 years were included in a phase II clinical trial with DTX. The dose given to them was 100 mg/m2 each 3 weeks. A total of 8 patients (in 33 evaluable) experienced partial remission for a period of about 7.6 months. On the other hand, 11 patients had their disease stabilized with this approach. This concludes that docetaxel is an active agent in advanced gastric cancer (Sulkes et al., 1994).

CTX was used for treating various solid tumors (Tsao et al., 2014b). Concerning the case of metastatic castration-resistant prostate cancer, a dose of cabazitaxel of 10–25 mg/m^2^ allowed the achieving of a median survival of 15.1 months (Bahl et al., 2013).

## 6 Pharmacokinetics, limitations, and strategies to increase the bioavailability and efficacy of taxanes

### 6.1 Pharmacokinetics of taxanes

In humans, PTX exhibits pharmacokinetics that are non-linear. This means that changes in dosage result in disproportionate alterations in both the peak paclitaxel concentrations in plasma and the areas under the curve when compared to the time profile. Many pharmacokinetics models do not succeed in truly describing the disposition of paclitaxel in the body of human beings. Although the safety profile of the drug is generally good, in cases of neutropenia, the concentration of paclitaxel in plasma frequently exceeds the threshold values, for example. Scientists and researchers are still making efforts to define the disposition of the paclitaxel drug along with its therapeutic efficacy (Kearns et al., 1995). The bioavailability of paclitaxel has been enhanced by coupling it with P-glycoprotein inhibitor KR30031. Analogously, the administration of paclitaxel with ketoconazole increased the bioavailability of the drug by about 1.60–1.7 fold per comparison with the controls (Woo et al., 2003).

CTX displays triphasic kinetics, with each phase having a distinct half-life. Although it has a good safety profile, certain health-related issues have been reported over time, including a high frequency of hematologic toxicity and infectious diseases. Additionally, some gastrointestinal issues such as vomiting, nausea, diarrhea, and constipation have been reported (Tsao et al., 2014b).

DTX exhibits low bioavailability, which is mainly attributed to its affinity for binding to the P-glycoprotein and its metabolism by cytochrome P450 (CYP3A4) in the liver. Experimental works showed that coupling docetaxel with cyclosporine (P-glycoprotein inhibitor and CYP3A4 substrate) increased docetaxel bioavailability by 90% (Malingré et al., 2001). The pharmacokinetics of docetaxel indicate that the plasma-concentration curve increases proportionally with the dose. Additionally, it should be noted that the clearance of docetaxel becomes more challenging with increasing age. This is why older patients should receive a reduced dosage of the drug (Clarke and Rivory, 1999).

### 6.2 Strategies to increase bioavailability of taxanes

#### 6.2.1 Nanoformulations

**FIGURE 3:**
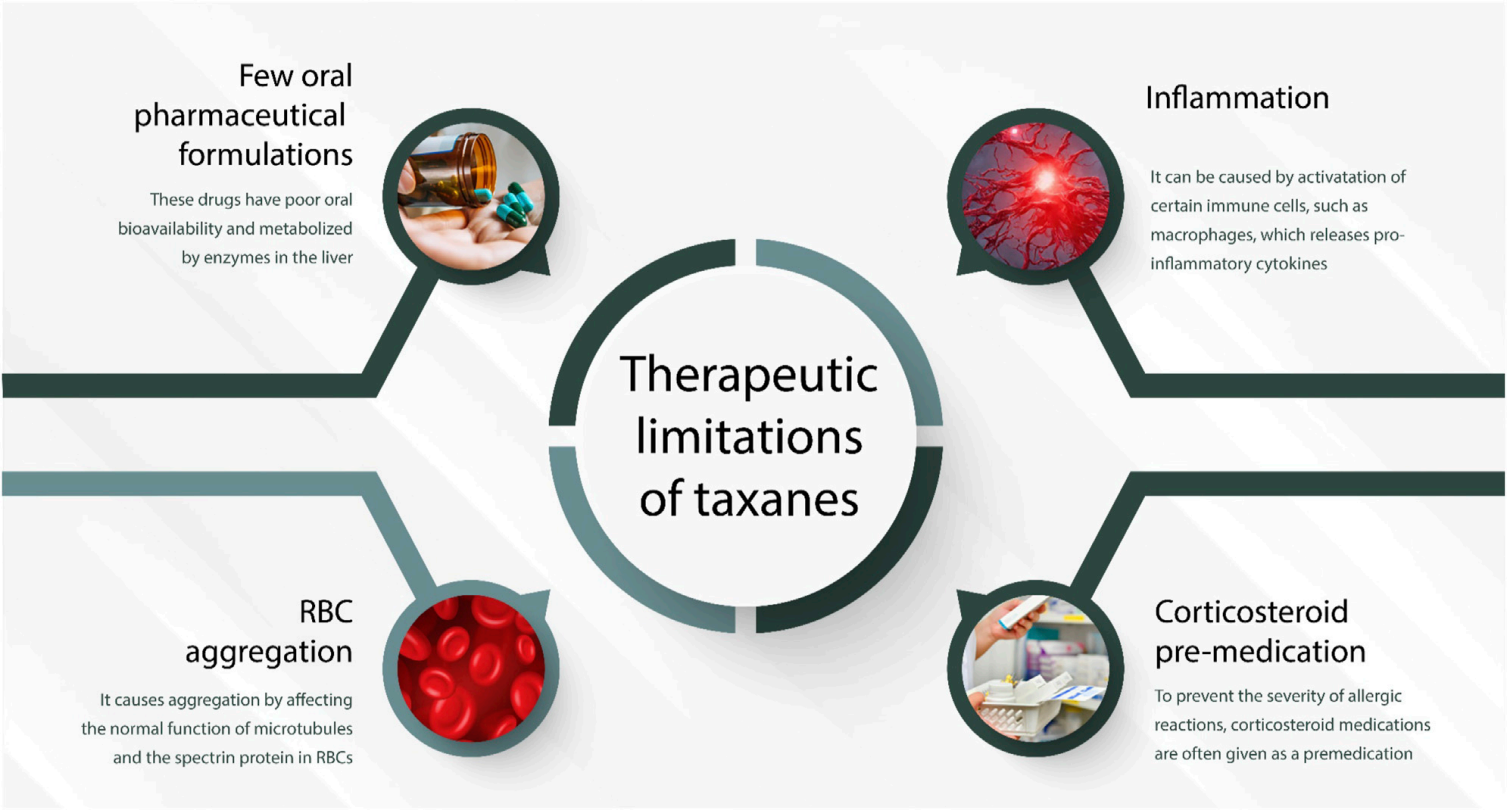
Summarized scheme regarding the most representative therapeutic limitation of taxanes in medical oncology. Abbreviation: red blood cell, RBC.

Paclitaxel is administered intravenously and has low water solubility. This explained why the first commercial formulation included a vehicle of non-ionic surfactant (polyoxyethylated castor oil—Cremophor EL). Although this increased the bioavailability of the drug, it did it at the expense of side effects like hypersensitivity reactions, increased erythrocyte aggregation, and peripheral neuropathy (Gelderblom et al., 2001) (Figure 3). Nanotechnological PTX vehicles have emerged with the dominance of nanoparticle albumin-bound-(nab-) PTX. In addition to increasing solubility and decreasing adverse reactions, this albumin nanoparticle activates Gp60 albumin-specific receptors on endothelial cells, leading to increased endothelial binding, transcytosis, and increased drug concentration in neoplastic tissues (Desai et al., 2006b). On the other hand, the low-hypersensitivity reactions with nab-PTX infusion in comparison with diluted polyoxyethylated castor oil PTX excluded the need for corticosteroid pre-medication and/or treatment, a fact that prunes the nab-PTX as a more attractive agent to combine with immune-modulators, as is the case of the PD-1 inhibitors in lung or breast cancer. Liposome-entrapped PTX (LEP-ETU), when compared with PTX formulation with polyethoxylated castor oil, displayed a similar pharmacokinetic profile, but it was associated with less toxicity and an overall similar efficacy. Liposomal entrapment technologies allow electrically charged lipids to interact with a drug (with an opposite charge), creating a carrying formulation. Generally, the benefits in terms of reduced adverse events along with the administration of the drug, better bioavailability and tolerability allow the administration of higher cumulative doses of entrapped PTX. As (nab-) PTX, LEP-ETU does not generally require corticosteroids as pre-medication (Slingerland et al., 2013).

Genexol PM is a polymeric micelle formulation of paclitaxel approved for the treatment of non-small-cell lung carcinoma as well as breast cancer. It is a low-molecular di-block co-polymer. Genexol PM has a lower toxicity profile than the other Taxol derivatives. Genexol PM is mainly used for chemoradiation therapy for NSLC (Werner et al., 2013). The NK-105 is a micelle nanoparticle formulation of paclitaxel. It enhances the anti-tumor activity of paclitaxel through self-association. PTX is found in the inner core of the micelle system through hydrophobic interactions with the drug as well as the co-polymer of PTX. The AUC (area under the curve) values were higher for the NK-105 than for paclitaxel. Experimentation has proved that NK-105 has more potent anti-tumor activity in the cancer cell line Ht-29 per comparison with paclitaxel alone, also displaying a reduced neurotoxic potential (Hamaguchi et al., 2005).

SB05 is a liposomal formulation of about 200 nm in diameter. It is formed from the cationic phospholipid DOTAP as well as neutral DOPC in the molar ratio of 53:47. Currently, phase III trials are being conducted for SB05 for triple-negative breast cancer patients (Sofias et al., 2017).

#### 6.2.2 Oral formulations

The common oral formulations of taxanes are Oraxol, DHP107, and ModraDoc001—administered at a fixed dose. Most of them are formulated with a polymer that can easily dissociate in plasma, thus maintaining plasma concentrations for a longer duration. However, adverse reactions such as nausea, fatigue, gastrointestinal issues, and neurotoxicity have been reported. The other limitations include the bioavailability and safety profiles as well as the coupling of inhibitors with the formulations.

##### 6.2.2.1 Oraxol

Oraxol is an oral formulation of paclitaxel administered with a novel, minimally absorbed P-glycoprotein inhibitor, Encequidar (HM30181A). This phase Ib study was conducted to determine the maximum-tolerated dose (MTD) of Oraxol administered at a fixed dose for up to 5 consecutive days in patients with advanced malignancies. There were certain side effects like fatigue, nausea, vomiting, and neutropenia. However, no hypersensitive reactions were observed. Out of 28 patients, only two achieved a partial response, and 18 were able to attain a stable disease. Overall, the oral administration was considered safe and depicted anti-tumor activity (Ma W. W. et al., 2022).

##### 6.2.2.2 DHP 107

DHP 107 is a novel formulation for oral paclitaxel, which has the mucoadhesive lipid-free Cremophor ElL. Administering DHP 107 orally results in enhanced absorption and tissue distribution of paclitaxel without the need to couple the administration with P-gp inhibitors. DHP 107 is a mucoadhesive lipid that enhances the drug delivery process as well as absorption in the epithelial cells of the intestinal tract (Hong et al., 2013).

##### 6.2.2.3 ModraDoc001

The ModraDoc001 (DTX) is a solid dispersion formulation hydrophilic polymer. This particular formulation significantly enhances the rate of dissolution along with the extent of the pharmaceutical ingredient of interest. The ModraDoc001 has been synthesized through the freeze–drying method. The most suitable carrier for ModraDoc001 is polyvinylpyrrolidone K-30. The surfactant is necessary for increasing the dissolution rate of docetaxel. The pharmacokinetics showed that the solid dispersion formulation had better plasma concentrations than the simple docetaxel drug (Jibodh et al., 2013).

## 7 Limitations

PTX and its various formulations possess certain limitations, with hypersensitivity reactions being a primary concern. Additional adverse effects associated with paclitaxel include peripheral neuropathy, myalgias, neutropenia, and arthralgia. These side effects can cause significant discomfort and complications for patients undergoing treatment. In recent years, advancements in nanoparticle technology have provided protection against surfactant or solvent-related adverse reactions. PTX is known for its poor solubility, necessitating its combination with a solvent or surfactant during the manufacturing process. The development of nanoparticle formulations has mitigated some of the side effects related to these added components. Furthermore, the administration of H2 blockers has been shown to be effective in minimizing the risk of life-threatening hypersensitivity reactions. This approach has been instrumental in improving the safety profile of paclitaxel-based therapies for patients. Despite these improvements, it is essential to recognize that both traditional and nanoparticle formulations of PTX can still cause side effects such as hair loss, muscle pain, and joint pain (Figure 3). These side effects may be experienced by patients regardless of the specific formulation utilized (Naganuma et al., 2019).

## 8 Conclusion

Taxol derivatives disrupt the microtubule assembling dynamics, arrest the cell cycle at the G2/M phase, and trigger apoptosis (Kampan et al., 2015). Such properties led to increased awareness about the potential of these derivatives in neoplastic disorders. This was confirmed by multiple clinical trials which unveiled their potential in pre-defined settings that encompassed solid malignancies. The journey to a sustainable and economical manufacturing process will ultimately lead to a decrease in the production and distribution time and cost (making it easier to access and afford these drugs worldwide), either by empowering semi-synthetic processes or the usage of genetically engineered bioreactors (Flores-Bustamante et al., 2010). Given the transitional phase of oncology that we are facing today, a shift from classical chemotherapy to immunomodulatory drugs and precision medicine has posed a challenge to the repurposing of taxanes and their refinement. The successful combination of PTX with PD-1 inhibitors was only possible due to the development of different formulations that shared a similar pharmacokinetic profile. These formulations displayed fewer infusion-related adverse reactions than PTX formulated with polyethoxylated castor oil, thereby sparing the use of corticosteroids (Slingerland et al., 2013) that would, otherwise, partially abrogate the desired immunological stimulus. The pathophysiological basis of cancer burden and the mechanism of action of taxanes raise awareness toward the potential risk for secondary neoplasms. For instance, Taxol derivatives are known to induce therapy-related myelodysplasia (t-MDS) or give rise to treatment-related acute myeloid leukemia (t-AML) as a toxicity side effect. The hematological neoplasms generally present with a complex karyotype (translocations involving chromosome 16 are frequent), a fact that, pathophysiologically, agrees with the blockage of the microtubular dynamics induced by Taxol derivatives in cellular populations with higher turnover, as myeloid cells. Herein, a putative co-existence of defective DNA damage repair mechanism, together with the therapy-specific DNA damage, may explain the role of Taxol derivatives in t-SMD/t-AML that arise from cellular populations with an incredibly higher turnover (Smith, 2003). To date, we are deciphering the genomic landscape of cancer. According to epidemiological data, several malignancies are associated with germline variants affecting DNA damage repair in a proportion that, for example, averages from 10% of breast cancer cases up to 14–18% of ovarian cancer cases (Jiang et al., 2020). Considering that such defects are not routinely searched in every patient and the regulome of the DNA repair machinery is not fully understood, it is fair to speculate that inhibiting the microtubular depolymerization process in highly proliferating non-neoplastic cells could potentially lead to therapy-related malignancies. Although some of these cells do not repair the inflicted damage, they may exhibit a transcriptional program that enables them to escape apoptosis and proliferate in the form of an aberrant clone, leading to the development of neoplasms such as myeloid malignancies. The germline DNA damage repair machinery defects in oncological patients will potentiate the chemotoxic effects of specific chemotherapeutic agents. The need to search for these germline variants in oncological patients is a pressing question that will be imposed in the not-too-distant future, in line with targeted and precision medicine approaches.

## Abbreviations

U.S.A., United States of America; F.D.A, Food and Drug administration; TNF, tumor necrosis factor; IL, interleukin factor; NOS, nitric oxide synthase; COX-2, cyclooxygenase 2; PTX, paclitaxel; TLR, toll-like receptor; T-Regs, T-regulatory cells; CD-95, cluster of differentiation 95; HIF, hypoxia-inducible factor; EMT, epithelial-to-mesenchymal transition; TGF, tumor growth factor; DAB, deacetyl baccatin; MBC, metastatic breast cancer; HER, human epidermal growth factor; OS, overall survival; mTNBC, metastasized triple-negative breast cancer cells; PD-L1, programmed death ligand-1; ITT Group, intention to treat; TNT, triple negative trial; PFS, progression-free survival; BEP, bleomycin, etoposide, cisplatin; t-MDS, therapy-related myelodysplasia; t-AML, therapy-related acute myeloid leukemia.
